# Hepatic pyruvate carboxylase expression differed prior to hyperketonemia onset in transition dairy cows

**DOI:** 10.1371/journal.pone.0241929

**Published:** 2020-11-09

**Authors:** Kristina A. Weld, Rafael Caputo Oliveira, Sandra J. Bertics, Sophia J. Erb, Heather M. White

**Affiliations:** Department of Dairy Science, University of Wisconsin – Madison, Madison, Wisconsin, United States of America; Michigan State University, UNITED STATES

## Abstract

Fatty acids (**FA**) provide an energy source to the liver during negative energy balance; however, when FA influx is excessive, FA can be stored as liver lipids or incompletely oxidized to β-hydroxybutyrate (**BHB**). The objectives of this study were to quantify plasma and liver FA profiles and hepatic gene expression in cows diagnosed with hyperketonemia (**HYK**; BHB ≥ 1.2 m*M*) or not (**nonHYK**; BHB < 1.2 m*M*) to determine a relationship between FA profile and expression of hepatic genes related to oxidation and gluconeogenesis. Production parameters, blood samples (-28, -3, 1, 3, 5, 7, 9, 11, and 14 d relative to parturition; n = 28 cows), and liver biopsies (1, 14, and 28 d postpartum; n = 22 cows) were collected from Holstein cows. Cows were retrospectively grouped as HYK or nonHYK based on BHB concentrations in postpartum blood samples. Average first positive test (BHB ≥ 1.2 m*M*) was 9 ± 5 d (± SD). Cows diagnosed with HYK had greater C18:1 and lower C18:2 plasma proportions. Liver FA proportions of C16:0 and C18:1 were related to proportions in plasma, but C18:0 and C18:2 were not. Some interactions between plasma FA and HYK on liver FA proportion suggests that there may be preferential use depending upon metabolic state. Cows diagnosed with HYK had decreased pyruvate carboxylase (***PC***) expression, but no difference at 1 d postpartum in either cytosolic or mitochondrial isoforms of phosphoenolpyruvate carboxykinase (***PCK***). The increased *PC* to *PCK* ratios in nonHYK cows suggests the potential for greater hepatic oxidative capacity, coinciding with decreased circulating BHB. Interestingly, FA, known regulators of *PC* expression, were not correlated with *PC* expression at 1 d postpartum. Taken together, these data demonstrate that HYK cows experience a decrease in the ratio of hepatic *PC* to *PCK* at 1 day postpartum prior to HYK diagnosis which, on average, manifested a week later. The differential regulation of *PC* involved in HYK diagnosis may not be completely due to shifts in FA profiles and warrants further investigation.

## Introduction

During the transition period, negative energy balance (**EB**) occurs when a cow’s energy intake is insufficient to meet the added energy requirements of lactation [1]. Fatty acids (**FA**) are mobilized from adipose tissue to provide an energy source which is used for maintenance of extra-mammary tissues and sparing of glucose for lactose synthesis. Additionally, hepatic oxidation of FA can provide energetic support for gluconeogenesis, and the triglyceride (**TG**) glycerol backbone can serve as a gluconeogenic precursor. Excessive FA uptake by the liver can overwhelm hepatic oxidative capacity which leads to acetyl-CoA from β-oxidation undergoing either ketogenesis or FA undergoing re-esterification in the liver to be stored as TG rather than being completely oxidized through the TCA cycle [2]. The result of increased ketogenesis and insufficient peripheral tissue uptake of ketone bodies is hyperketonemia (**HYK**), a metabolic disorder associated with numerous negative health and production outcomes [3–5]. It has been proposed that the relative cataplerosis of oxaloacetate out of the TCA cycle may result in decreased TCA cycle capacity for complete oxidation of acetyl-CoA, thus increasing carbon flux through ketogenesis and storage of lipids [6, 7]. The relative gene expression ratio of pyruvate carboxylase (***PC***), a key enzyme that influences supply of the oxaloacetate pool, to phosphoenolpyruvate carboxykinase (***PCK***), an enzyme that commits intermediates to gluconeogenesis, may provide insight into the balance of these pathways.

Although the general etiology of HYK has been described, the effects of HYK status on nutrient metabolism, specifically the potential for preferential FA use for hepatic fates, are not fully understood. The primary non-esterified fatty acid (**NEFA**) circulating in plasma during negative EB are C16:0, C18:0, and C18:1, which are derived from adipose tissue depots where they are stored [8–10]. Compared to cows restricted to maintenance energy intake prepartum, cows fed above maintenance requirements prepartum to induce fatty liver had increased blood NEFA and increased C16:0 and C18:1 in plasma and liver postpartum [8]. Similarly, cows diagnosed with HYK lost more body condition score (**BCS**) during the transition period than cows not diagnosed with HYK (**nonHYK**) [11] and have greater NEFA concentrations [4]. Given that *PC* has been demonstrated to be regulated by differences in FA concentration [12] and is greatest postpartum when circulating NEFA are highest [13], hepatic gene expression may be influenced by the above noted changes in FA profile. To date, differential FA profiles have not yet been demonstrated to impact *PC* expression in vivo; however, understanding how FA and other metabolites may impact capacity for complete oxidation or storage of FA postpartum is of interest.

Although changes in plasma and liver FA profiles have been previously demonstrated in cows exposed to different prepartum diets to induce metabolic disorders postpartum [8], the goal of this study was to determine if these differences occur in cows with similar prepartum diets and to further our understanding of these effects by simultaneously examining the expression of *PC* and *PCK*, key hepatic genes involved in gluconeogenesis and TCA cycle oxidation. The hypothesis of this research is that changes in plasma and liver FA profiles are reflective of adipose tissue mobilization and that shifts in FA profile and amount in cows with HYK would have downstream regulatory effects on hepatic gene expression. We therefore had three primary objectives in the current research: 1) to quantify plasma and liver FA profiles; 2) to quantify hepatic *PC* and *PCK* gene expression in cows diagnosed with HYK or not; and 3) determine if there is a relationship between circulating FA profiles and hepatic gene expression between HYK and nonHYK cows.

## Materials and methods

All animal protocols were approved by the University of Wisconsin − Madison College of Agricultural and Life Sciences Animal Care and Use Committee.

### Animals and diets

Forty Holstein cows in second or later lactations were housed at the Dairy Cattle Center at the University of Wisconsin—Madison, Madison, WI in tie stalls on rubber mats from 28 days prior to calving until 45 days postpartum between June and October 2016. Cows were fed once daily, milked twice daily (0530 and 1730 h), and had ad libitum access to water. Cows were fed a high energy diet prepartum (partial mixed ration + 5.25 kg of cracked corn) and began dietary treatments postpartum (Table 1).

**Table 1:** Ingredients of diet offered and chemical composition of prepartum partial mixed ration (PMR), topdressed cracked corn, and postpartum control and fermented ammoniated condensed whey (FACW) lactation diets.

	Prepartum^2^		Lactation	
Item (% DM ± SD)^1^	PMR	Cracked corn	Control	FACW
Ingredient offered	−	−		
Corn silage	45.0 ± 1.32	−	31.5 ± 0.98	31.6 ± 0.95
Alfalfa Silage	−	−	23.1 ± 1.66	23.1 ± 1.72
Cottonseed, fuzzy	−	−	6.0 ± 0.23	6.0 ± 0.21
Corn ground shell	−	−	17.4 ± 0.37	17.3 ± 0.44
Soybean meal	31.7 ± 0.76	−	9.7 ± 0.20	7.0 ± 0.18
Expeller soybean meal^3^	−	−	4.9 ± 0.10	4.8 ± 0.12
Soy hulls	−	−	3.9 ± 0.08	3.7 ± 0.09
FACW^4^	−	−	−	2.9 ± 0.07
Prepartum Mix^5^	5.0 ± 0.12	−	−	−
Postpartum Mix^6^	−	−	3.5 ± 0.07	3.6 ± 0.09
Straw	18.2 ± 0.45	−	−	−
Nutrient composition				
DM	47.0 ± 0.52	87.5 ± 0.59	54.9 ± 1.32	54.6 ± 1.45
CP	19.3 ± 1.35	8.4 ± 0.20	16.9 ± 0.29	17.1 ± 0.21
ADF	22.7 ± 0.46	2.6 ± 0.51	21.0 ± 0.96	20.7 ± 0.83
NDF	33.7 ± 0.47	7.2 ± 0.23	27.9 ± 0.74	27.4 ± 1.01
Starch	20.3 ± 2.03	70.3 ± 1.44	26.7 ± 1.44	26.3 ± 1.07
Ether extract	2.2 ± 0.11	4.1 ± 0.36	4.1 ± 0.16	4.5 ± 0.16
Ash	10.3 ± 0.53	1.4 ± 0.17	7.8 ± 0.27	7.9 ± 0.19
Water-soluble carbohydrates	5.9 ± 0.48	3.9 ± 0.37	4.4 ± 0.61	4.9 ± 0.53
NFC	37.0 ± 1.92	80.5 ± 0.20	44.7 ± 0.48	44.5 ± 0.71
NE_L_ 3x (Mcal/kg diet DM^7^)	1.52 ± 0.02	2.08 ± 0.01	1.67 ± 0.01	1.69 ± 0.02

^1^Values presented represent the average of the dietary components after being adjusted for DM based on a weekly sample and analysis is based off averages of monthly composites from weekly samples

^2^Cows were fed partial mixed ration (**PMR**) ad libitum and 5.25 kg of top-dressed cracked corn

^3^Exceller meal (Quality Roasting Inc., Valders, WI).

^4^GlucoBoost (Fermented Nutrition Corporation, Luxemburg, WI; 72.7% DM Lactic acid, 55.5% CP).

^5^Prepartum mix: CaCO_3_ (32.1%), CaH_4_P_2_O_8_ (18.19%), CaSO_4_ (14.7%), NaCl (8.78%), MgO (7.5%), MgSO_4_ (14.7%), mineral oil (0.6%), selenium yeast 2000 (0.5%, Prince Agri Products, NJ), Rumensin-90 (0.3%, Elanco Animal Health, Greenfield, IN), biotin (0.3%, DSM Nutritional Products, Belvidere, NJ), vitamin A (278.4 KIU/kg), vitamin D_3_ (84.1 KIU/kg), and vitamin E (4058 IU/kg).

^6^Postpartum mix: CaCO_3_ (33.5%), NaHCO_3_ (30.5%), grease (12.2%), Dical 21% (6.1%, Sanimax, DeForest, WI), MgO (6.1%), NaCl (9.9%), ZnO (0.3%), MnO (0.3%), CuSO_4_ (0.2%), selenium yeast 2000 (0.1%, Prince Agri Products, NJ), mineral oil (0.1%), FeSO_4_ (55100 ppm), E.D.D.I. 99% (5510 ppm, IodiTech Inc., Kansas City, MO), CoCO_3_ (1624 ppm), vitamin A (224.7 KIU/kg), vitamin D_3_ (44.9 KIU/kg), vitamin E (1123 IU/kg), Rumensin-90 (0.2%, Elanco Animal Health, Greenfield, IN), and biotin (0.1%, DSM Nutritional Products, Belvidere, NJ).

^7^ Net Energy of Lactation; Estimated with the NRC (2001) [14] equations to calculate NE_L_ at 3x maintenance.

Postpartum diets were identical except for addition of a fermented ammoniated condensed whey (**FACW**) supplement (GlucoBoost; Fermented Nutrition Corporation, Luxemburg, WI; Table 1) to the treatment diet. A companion paper, Caputo Oliveira et al. (2019) [15], contains details on experimental design, diet formulation and chemical composition, data collection, and animal handling. For the objectives of this analysis, a subset of the cows retrospectively classified as either HYK or nonHYK were evaluated. Classification as HYK was determined as plasma β-hydroxybutyrate (**BHB)** ≥ 1.2 m*M* thereby including sub-clinical and clinical cases of HYK and clinical symptoms were not required as inclusion criteria. Twenty-eight cows (n = 28) were chosen in which to analyze FA profiles of plasma (HYK: [BHB] ≥ 1.2 m*M* on at least one d of testing, n = 13; nonHYK: plasma BHB < 1.2 m*M*, n = 15) representing 14 cows from each of the postpartum dietary treatments (+ FACW: HYK n = 4, nonHYK n = 10; − FACW: HYK n = 9, nonHYK n = 5). Within this cohort, a subset was randomly chosen for additional liver tissue analysis (HYK n = 10; nonHYK n = 12). Cows classified as HYK had 7.1 ± 9.0 days above the threshold (no difference by dietary treatment). Cows were treated for HYK only if they reached a BHB ≥ 3.0 m*M*.

### Sample collection and analysis

Daily dry matter intake (**DMI**) was determined by recording feed offered and refused. Feed ingredients were sampled weekly. Samples were dried at 55°C for 48 h, ground through a 1 mm screen (Wiley Mill; Arthur H. Thomas, Philadelphia, PA), and analyzed for chemical composition by Dairyland Labs (Arcadia, WI). Milk yield was measured at each milking and composite milk samples taken at two consecutive milkings each week to be analyzed for composition by Agsource Cooperative Services (Menomonie, WI).

Body weights (**BW**) and BCS were measured at 28 and 14 d prepartum and 1, 14, and 28 d postpartum. Body condition score was measured by two trained personnel using a five-point scale with quarter-point increments [16] and averaged within timepoint. Blood samples were collected from the coccygeal vessels at 28 and 3 d prepartum and 1, 3, 5, 7, 9, 11, and 14 d postpartum. Blood was drawn into vacutainer tubes containing sodium fluoride and potassium oxalate (BD Vacutainer; Becton, Dickinson and Company, Franklin Lakes, NJ) after the morning milking (0600h), placed on ice, centrifuged at 2,000 × *g* for 15 minutes, and aliquots of plasma stored at -20°C until analysis. Select plasma samples were analyzed for NEFA concentration (1, 3, 5, 7, 11, 14 DRTC; Wako NEFA-HR(2) Microtiter Procedure kit; Wako Diagnostics, Richmond, VA), BHB (all postpartum samples; Stanbio BHB LiquiColor kit Procedure number 2440–058; Stanbio Laboratory, Boerne, TX), and FA profile (-28, -3, 1, 3, 7, 11, and 14 DRTC). Fatty acid profile was determined by acid methylation using 5% methanolic HCl and chloroform in a 70°C water bath for 2 h followed by neutralization with K_2_CO_3_ [17]. Chloroform was removed and evaporated under nitrogen to a volume of 100 μL which was analyzed by GC. Fatty acids were determined on a PerkinElmer Clarus 680 GC (PerkinElmer, Norwalk, CT) using column specifications and temperature program described by Weld and Armentano [18]. Peaks were identified based on commercially available individual FAME mixtures: FIM-FAME-5, -6, and -8 (4210, 2009, 2012; Matreya Inc., Pleasant Gap, PA) and analyzed using the TotalChrom Workstation V 6.3.2 software (PerkinElmer).

Liver samples were collected after administration of local subcutaneous anesthetic (10 mL of 2% lidocaine hydrochloride solution) via blind percutaneous biopsy at 1, 14, and 28 d postpartum using a 6 mm diameter outside, 4 mm diameter inside biopsy needle as described previously [19]. Liver was subsampled into empty RNA free tubes for future hepatic TG measurement or into RNA free tubes containing TRIzol (Life Technologies, Carlsbad, CA) for quantification of mRNA expression. All samples were flash frozen in liquid nitrogen and stored at -80°C until subsequent analysis. Liver TG were determined by extraction of TG [20] and quantification [21] as reported previously [19]. Hepatic TG FA profile was determined by acid methylation of a fraction of the extracted liver TG (after the methanol—chloroform extraction step) as described above for plasma FA.

Hepatic RNA was extracted in TRIzol (Life Technologies) and purified utilizing the Aurum Total RNA 96 Kit (Bio-Rad Laboratories, Hercules, CA) using a modified version of the manufacturer’s protocol. Total RNA of samples was quantified and quality assured (ratio of absorbance at 260 and 280 nm between 1.9 and 2.1) using a Synergy H1 Hybrid Spectrophotometer (BioTek, Winooski, VT). Further assurance of RNA was analyzed using a Bioanalyzer 2100 (Agilent; Santa Clara, CA), and the RNA integrity number of 7.0 indicated that RNA quality was sufficient for qPCR. One μg of purified RNA was reverse transcribed using iScript Reverse Transcription Supermix for Real Time-qPCR (Bio-Rad Laboratories, Hercules, CA) according to the manufacturer’s instructions in a C1000 Touch Thermo Cycler (Bio-Rad Laboratories). Gene expression was determined via Real Time-qPCR with SsoAdvanced SYBR (Bio-Rad Laboratories) in a CFX384 Real-Time System (Bio-Rad Laboratories). Primers used for gene expression quantification are reported in Table 2. Real Time-qPCR was done according to the following protocol: 30 s at 95°C and 45 cycles of 95°C and 55°C for 5 and 15 s, respectively. A melt curve starting at 65°C and increasing to 95°C at increments of 0.5°C every 5 s demonstrated a single end product of predicted size for each gene. Efficiency of all reactions were maintained between 90 and 110% based on the standard curve of a cDNA pool (comprised of equal quantities from each sample). Cycle data were transformed to starting quantity with Bio-Rad CFX Software (Bio-Rad Laboratories) utilizing the standard curve method as described in Weld et al. (2019) [22].

**Table 2:** Primers used for quantitative real-time PCR.

Gene^1^	GenBank accession	Position	Sequence (5’-3’)	Source
*GAPDH*	NM_001034034.2	Forward	AAGGTCGGAGTGAACGGATTC	[22]
		Reverse	ATGGCGACGATGTCCACTTT	
*RPL32*	NM_001034783.2	Forward	AGACCCCTCGTGAAGCCTAA	[22]
		Reverse	CCGCCAGTTCCGCTTGATTT	
*RPS9*	NM_001101152.2	Forward	CCTCGACCAAGAGCTGAA	[23]
		Reverse	CCTCCAGACCTCACGTTT	
*UBB*	NM_174133.2	Forward	TGGCATTGTTGGGTTCCTGT	Verified within
		Reverse	CGAAGATCTGCATTTTGACCTGT	
*ACTB*	NM_173979.3	Forward	GCGTGGCTACAGCTTCAC	Verified within
		Reverse	TTGATGTCACGGACGATTT	
*18S*	NR_036642.1	Forward	ACCCATTCGAACGTCTGCCCTATT	[24]
		Reverse	TCCTTGGATGTGGTAGCCGTTTCT	
*PC*	NM_177946.4	Forward	CCACGAGTTCTCCAACACCT	[25]
		Reverse	TTCTCCTCCAGCTCCTCGTA	
*PCK1*	NM_174737.2	Forward	AGGGAAATAGCAGGCTCCAGGAAA	[26]
		Reverse	CACACGCATGTGCACACACACATA	
*PCK2*	NM_001205594.1	Forward	CCATCATCTTTGGAGGCCGT	[22]
		Reverse	GACCTTCCCTTTGTGTTCAGC	

^1^*RPL32*: ribosomal protein L32; *RPS9*: ribosomal protein S9; *UBB*: ubiquitin B; *ACTB*: β-actin; *PC*: pyruvate carboxylase; *PCK1*: cytosolic phosphoenolpyruvate carboxykinase; *PCK2*: mitochondrial phosphoenolpyruvate carboxykinase

Abundance of six potential reference genes (18S, ribosomal protein L32 [*RPL32*], ribosomal protein S9, ubiquitin B, β-actin, and *GAPDH*) were quantified and tested for stability. Except for *RPL32*, potential reference genes had more than one cycle variation between the means (S1 Fig). Further, the combination of genes suggested by geNORM as the most stable (*GAPDH* and ribosomal protein S9) exhibited greater variation between the means compared with *RPL32*, although the effects of HYK and DRTC did not reach statistical significance. Thus, *RPL32* was selected to be utilized as the only reference gene and the genes of interest are expressed relative to it.

### Calculations and statistics

Analysis of DMI, milk production, BW, EB, BCS, hepatic gene expression, and plasma BHB, NEFA, and FA profile utilized 28 cows (HYK n = 13; nonHYK n = 15). A subset of these animals was included in the liver FA analysis (n = 22).

Energy balance was calculated as the difference between the daily energy intake of animals (DMI × diet Net energy of lactation [**NE**_**L**_]) and their requirements for maintenance and gestation or lactation pre- and postpartum, respectively. Requirements and milk energy were calculated according to the NRC (2001) [14].

Data were analyzed with the MIXED procedure of SAS 9.4 (SAS Institute Inc., Cary, NC). The model contained the fixed effects of HYK diagnosis, d relative to parturition, and their interaction. Cow within HYK observations were repeated across time and first order autoregressive used as the covariance structure. Block and cow within HYK diagnosis were always included in the random statement. Postpartum dietary treatment and its interaction with time were included in the random statement when they improved the model fit (AIC change > 5 units). When residuals were not normal, the variable was log transformed and is reported with the back-transformed means and 67% confidence interval. Significance was declared when *P* ≤ 0.05 and a tendency when 0.05 < *P* ≤ 0.10. When the effect of d was significant, the Tukey-Kramer adjustment was used to determine when differences between d were significant. When the interaction between d and HYK was *P* < 0.10, the SLICE option was utilized to determine significant effects of HYK within day. For variables without repeated measures (e.g. BCS change), the same model was used but without the effect of time or repeated measures. To determine the relationship between individual plasma and liver FA proportions, a regression equation was built in which HYK and plasma proportion were used to model the liver proportion. If the interaction was not significant, it was removed from the model and only the overall slope reported. The CORR procedure was used to characterize the relationships between the proportion of plasma and liver FA, between BCS change and FA proportions, and between plasma FA and liver gene expression using Pearson correlations. The number of paired observations available for specific correlations is given in the footnotes of tables. Significant differences between correlations were determined using a Z-test.

## Results

**Fig 1:**
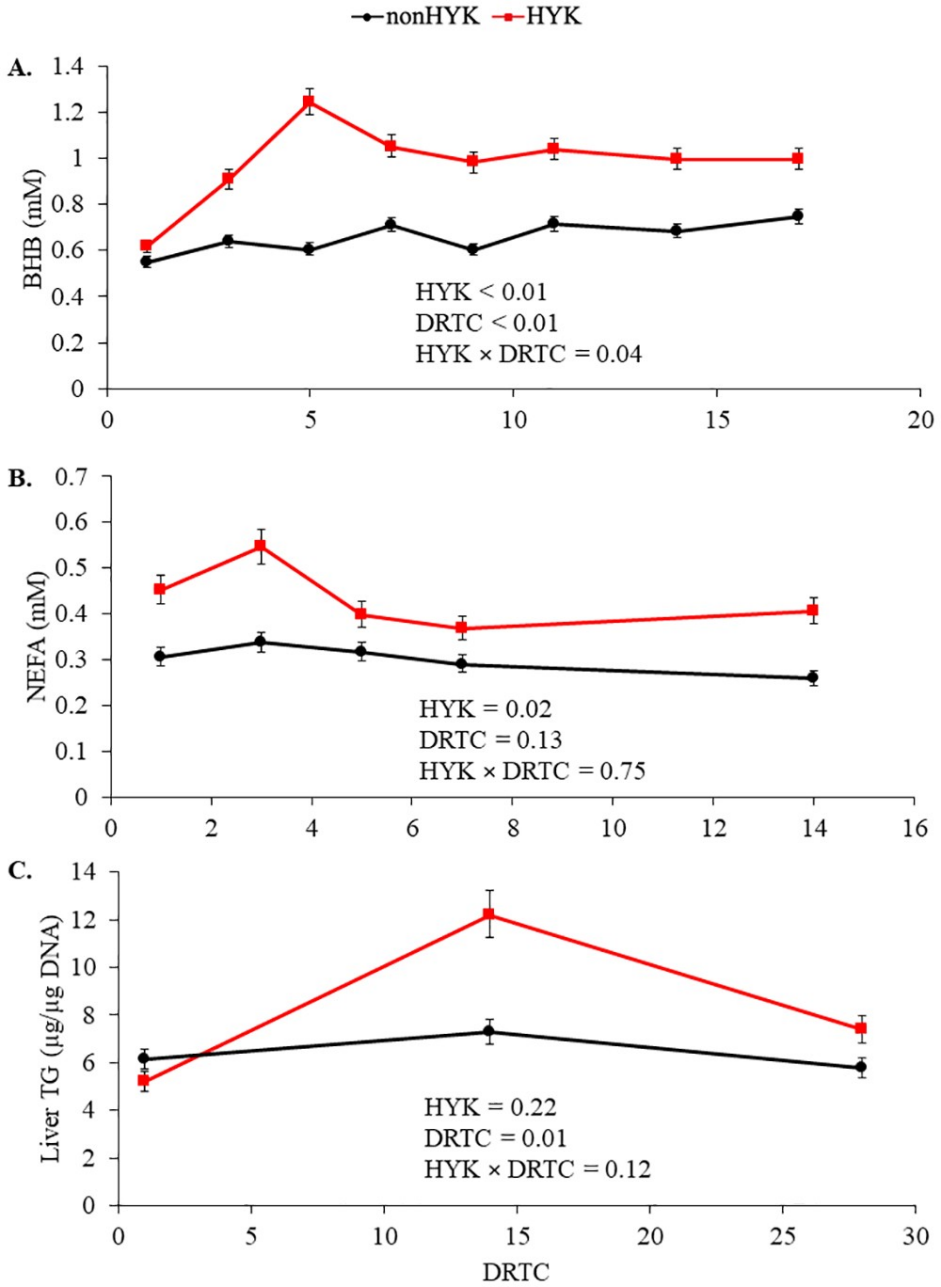
Blood metabolites and liver triglycerides of cows diagnosed as hyperketonemic or not. Postpartum β-hydroxybutyrate (BHB; A), non-esterified fatty acid (NEFA; B), and liver triglycerides (TG; C) in cows diagnosed as hyperketonemic (HYK; plasma BHB ≥ 1.2 mmol/L; red) or not (nonHYK; black). Variables were log transformed for analysis to achieve normal residuals and thus are reported as back-transformed means and a 67% confidence interval. Panel C: Effect of DRTC (*P* < 0.05) on liver TG denoted by differing superscripts for 1^b^, 14^a^, and 28^ab^ DRTC.

By definition, cows diagnosed with HYK had elevated plasma BHB postpartum (Fig 1A; *P* < 0.01) compared with their nonHYK counterparts and the interaction with time was significant (*P* = 0.04). The average first d of positive test (BHB ≥ 1.2 m*M*) was 9 ± 5 (± SD). Cows with HYK also had increased NEFA postpartum (Fig 1B; *P* < 0.02). Liver TG content was only numerically greater in HYK cows (Fig 1C; *P* = 0.22), and the interaction with time did not reach significance (*P* = 0.12). Overall changes in BW and BCS were not significantly different between HYK and nonHYK cows (Table 3; d × HYK *P* ≥ 0.17); however, the loss in BCS from 1 to 14 d postpartum was greater in cows diagnosed with HYK (*P* = 0.01). There was no significant difference in EB postpartum (*P* = 0.80) and no interaction with time (*P* = 0.15). The EB of HYK cows prepartum was less positive (*P* = 0.03) compared with nonHYK cows and there was no interaction with time (*P* = 0.61). Cows diagnosed with HYK had lower (*P* < 0.01) DMI prepartum and DMI tended (*P* = 0.09) to be lower postpartum; however, the decreased BW of HYK cows (*P* < 0.01) resulted in a lack of difference in DMI per BW between HYK and nonHYK cows (*P* ≥ 0.31). There was no evidence for a time by HYK diagnosis interaction on prepartum DMI (*P* ≥ 0.61). The amount of cracked corn provided daily was consistent, yet given the decreased prepartum DMI in cows diagnosed with HYK, those cows had an increased proportion of cracked corn in their prepartum diet compared to nonHYK (30.8 vs. 33.8 ± 0.1% DM; *P* = 0.03).

**Table 3:** Milk parameters, dry matter intake (DMI), body weight (BW), body condition score (BCS), and energy balance (EB) of cows diagnosed as hyperketonemic (HYK; plasma β-hydroxybutyrate ≥ 1.2 m*M*) or not (nonHYK; plasma β-hydroxybutyrate < 1.2 m*M*).

	LS means			*P*-Values		
Variable	nonHYK	HYK	SEM	HYK	time	HYK×time
Milk (kg/d)	43.6	41.7	1.8	0.45	<0.01	0.20
Milk (Mcal/d)	39.1	37.1	3.4	0.40	0.01	0.08
Milk Fat (kg/d)	2.44	2.32	0.21	0.45	0.24	0.03
Milk Protein (kg/d)	1.35	1.26	0.11	0.22	0.95	0.98
DMI (kg/d)						
Prepartum	16.9	14.8	1.0	<0.01	0.12	0.78
Postpartum	23.9	21.6	2.2	0.09	0.02	0.44
DMI/BW (%)						
Prepartum	2.11	2.03	0.12	0.31	0.05	0.54
Postpartum	3.05	3.04	0.28	0.96	<0.01	0.18
Milk/DMI (kg/kg)	1.91	2.09	0.09	0.15	0.61	0.27
EB (Mcal/d)^1^						
Prepartum	12.2	9.8	2.1	0.03	0.06	0.61
Postpartum	-11.5	-12.0	1.5	0.80	<0.01	0.15
BW (kg)	760	682	17	<0.01	<0.01	0.66
BCS	3.28	3.15	0.09	0.33	<0.01	0.17
BCS change						
-28 to 1 d	0.11	0.01	0.08	0.17	−	−
1 d to 14 d	-0.04	-0.24	0.05	0.01	−	−
14 d to 28 d	-0.08	-0.13	0.04	0.37	−	−

^1^NRC (2001) [14] equations used to calculate energy requirements for milk, maintenance (BW^0.75^ × 0.08), and gestation [(0.00318 × d of gestation—0.0352) × (calf body weight/45)]/0.218. Calculated NE_L_ of intake by multiplying the energy density of the diet by DMI.

**Fig 2:**
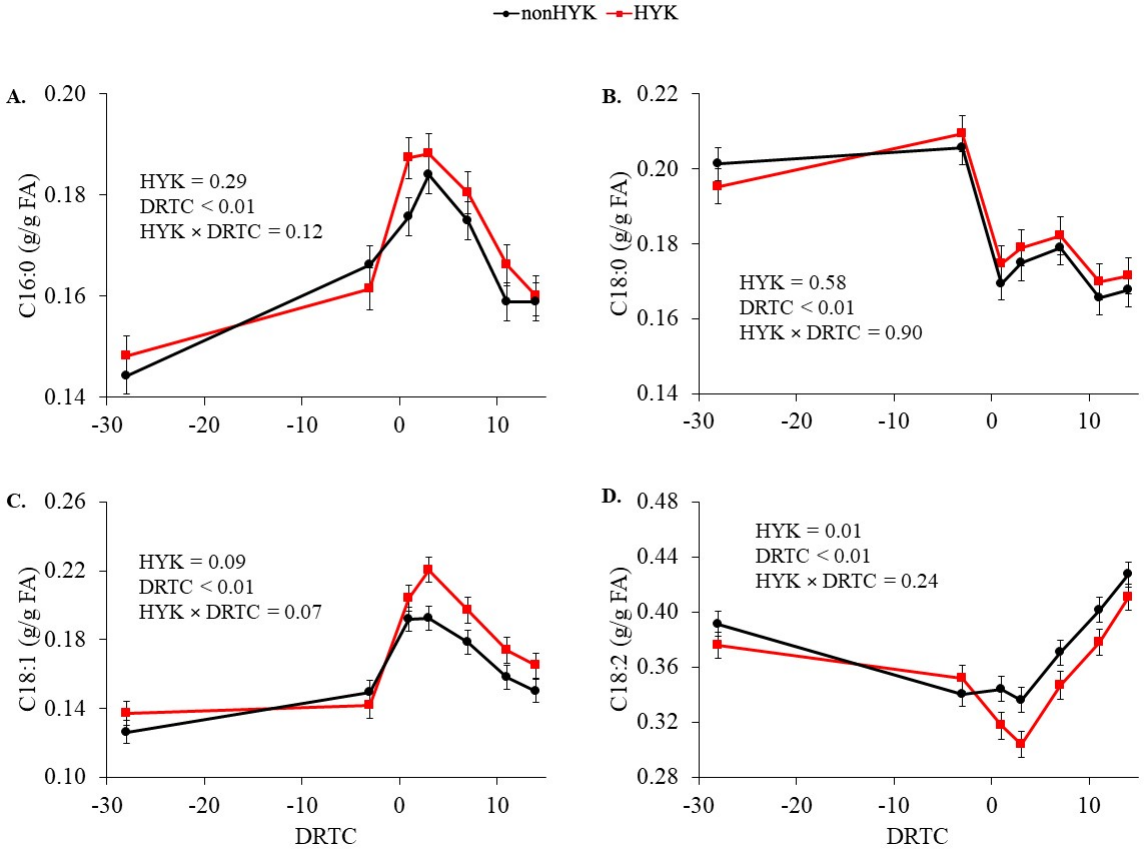
Peripartum plasma proportions of fatty acids in cows diagnosed as hyperketonemic or not. C16:0 (A), C18:0 (B), C18:1 (C), and C18:2 (D) fatty acids in cows diagnosed as hyperketonemic (HYK; plasma β-hydroxybutyrate ≥ 1.2 m*M*; red) or not (nonHYK; plasma β-hydroxybutyrate < 1.2 m*M*; black). Variables are reported using the SEM.

**Fig 3:**
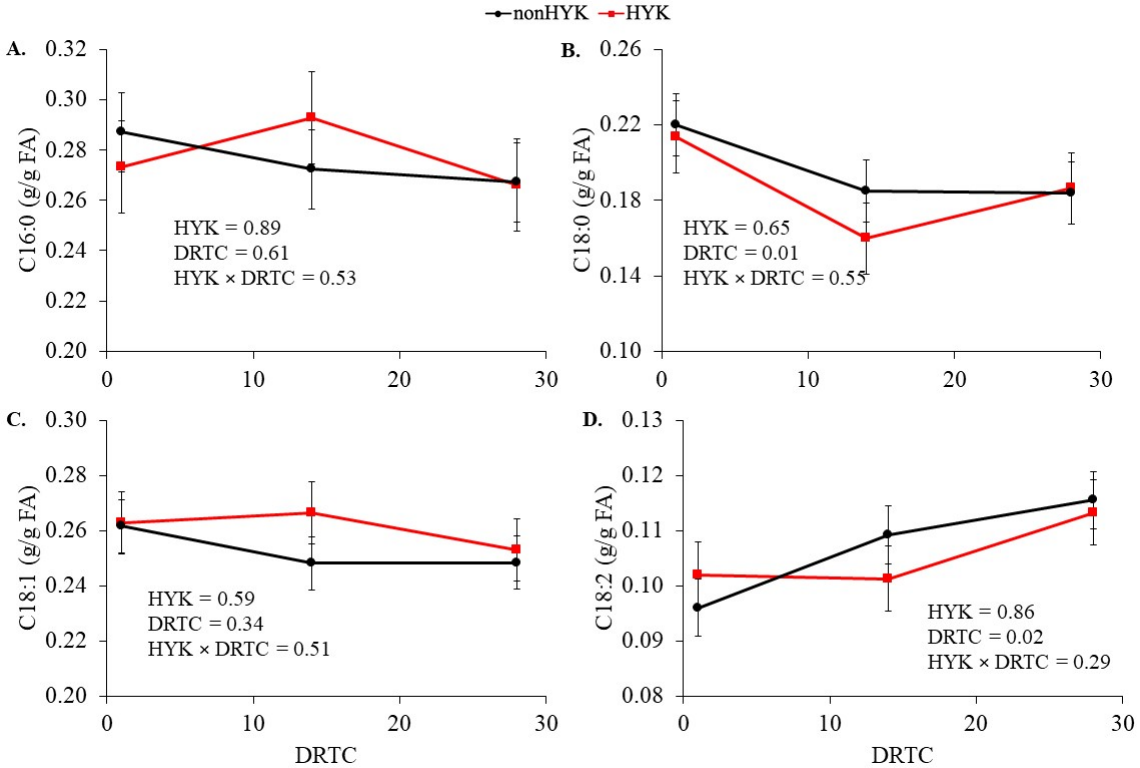
Postpartum liver proportions of fatty acids in cows diagnosed as hyperketonemic or not. C16:0 (A), C18:0 (B), C18:1 (C), and C18:2 (D) fatty acids in cows diagnosed as hyperketonemic (HYK; plasma β-hydroxybutyrate ≥ 1.2 m*M*; red) or not (nonHYK; plasma β-hydroxybutyrate < 1.2 m*M*; black) during the postpartum period. Variables are reported using the SEM.

**Fig 4:**
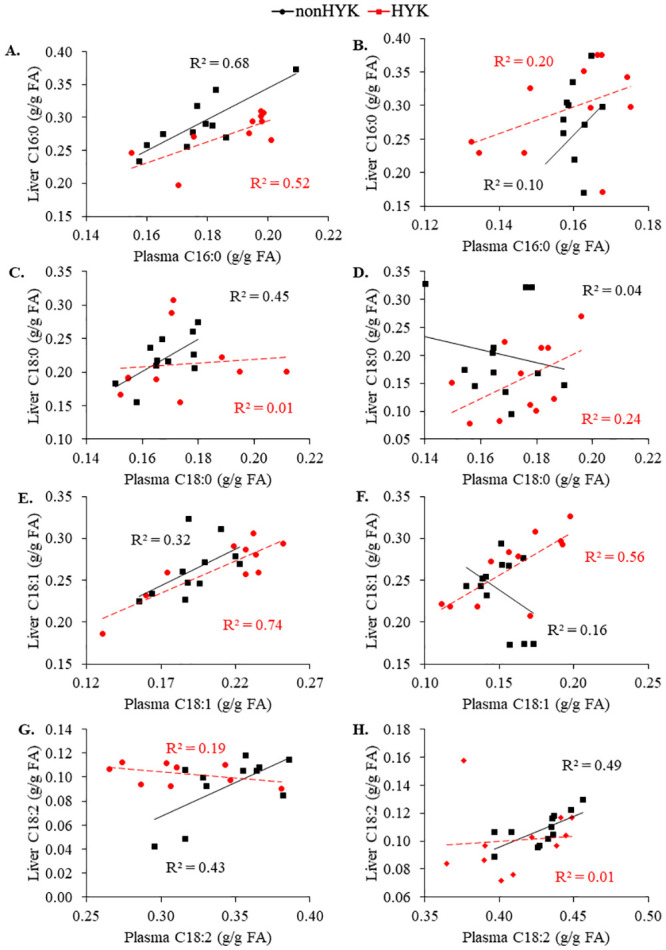
Correlations between plasma and liver proportions of fatty acids in cows diagnosed as hyperketonemic or not. C16:0 (A, B), C18:0 (C, D), C18:1 (E, F), and C18:2 (G, H) fatty acids at day 1 (left panels) and 14 (right panels) postpartum, in cows diagnosed as hyperketonemic (HYK; plasma β-hydroxybutyrate ≥ 1.2 mM; red) or not (nonHYK; plasma β-hydroxybutyrate < 1.2 m*M*; black).

At d 1 postpartum the proportion of plasma C16:0 and C18:1 increased (*P* < 0.05; Fig 2) and plasma C18:0 and C18:2 decreased (*P* < 0.05; Fig 2) compared with prepartum proportions. There were no effects of HYK on plasma C16:0 or C18:0 proportions (*P* ≥ 0.29; Fig 2). Time and HYK tended to interact (*P* = 0.07; Fig 2) to affect plasma C18:1 resulting in a significant increase in HYK cows at 3 d postpartum. The proportion of plasma C18:2 was decreased in HYK cows (*P* = 0.01). There were negative correlations between plasma C16:0 and C18:1 at 1 d postpartum and change in BCS postpartum in HYK cows (*P* ≤ 0.10; Table 4). Similarly, d 1 NEFA was highly positively correlated with plasma C16:0 and C18:1 and negatively correlated with plasma C18:2 (*P* < 0.05). Plasma C18:0 was not correlated with NEFA at d 1 (*P* > 0.10), although its proportion through 14 d postpartum was related to prepartum BCS (*P* < 0.05) in cows diagnosed with HYK. There were no significant effects of HYK on liver FA proportions (*P* > 0.59; Fig 3). The proportion of liver C16:0 and C18:1 on d 1 was related to the proportion in plasma independent of HYK status (*P* < 0.01; Table 5; Fig 4). The relationships between liver and plasma proportions of C18:1 differed between HYK and nonHYK cows on d 14 and between C18:2 proportions on d 1 (plasma proportion × HYK *P* = 0.01; Table 4). There was not a significant relationship (*P* = 0.31) between C18:0 proportions in plasma and liver on d 1 postpartum.

**Table 4:** Correlations of plasma fatty acid profile and non-esterified fatty acid (NEFA) with transition period body condition score (BCS) change in cows diagnosed with hyperketonemia (HYK; plasma β-hydroxybutyrate ≥ 1.2 m*M*) or not (nonHYK; plasma β-hydroxybutyrate < 1.2 m*M*)^1^.

	Correlations							
	C16:0		C18:0		C18:1		C18:2	
	nonHYK	HYK	nonHYK	HYK	nonHYK	HYK	nonHYK	HYK
NEFA (mmol/L)								
d 1	0.68*	0.76*	-0.08	0.06	0.74*	0.79*	-0.57*	-0.65*
d 14	-0.07	0.60*	0.11	0.02	0.16	0.68*	-0.13	-0.54^†^
ΔBCS -28 to 1								
d 1	-0.14	-0.52^†^	0.44	0.23	-0.14	-0.52^†^	-0.14	0.25
d 14	-0.15	-0.60*	0.29	0.25	0.07	-0.56*	-0.33	0.30
ΔBCS 1 to 14								
d 1	-0.72*	-0.30	0.12	-0.25	-0.59*	-0.35	0.42	0.44
d 14	-0.23	-0.06	-0.16	0.24	0.10	-0.16	-0.05	-0.02
ΔBCS 1 to 28								
d 1	-0.31	-0.40	0.08	-0.25	-0.01	-0.38	0.08	0.42
d 14	-0.48^†^	-0.25	-0.10	0.01	0.22	-0.48^†^	-0.12	0.14
ΔBCS 14 to 28								
d 1	0.52^†^	-0.23	-0.06	-0.08	0.67*	-0.15	-0.40	0.12
d 14	-0.29	-0.27	0.08	-0.30	0.13	-0.46	-0.08	0.23

^1^Number of paired observations per timepoint and group: nonHYK d 1 (n = 14); HYK d 1 (n = 12); nonHYK d 14 (n = 14), HYK d 14 (n = 12). Reported correlations are r-values from Pearson correlations.

*Indicates correlation is different than 0 (*P* ≤ 0.05)

^†^ Indicates correlation is different than 0 (0.05 < *P* ≤ 0.10)

**Table 5:** Regression coefficients for the slope of the relationship between individual liver fatty acid and plasma fatty acid in cows diagnosed with hyperketonemia (HYK; plasma β-hydroxybutyrate ≥ 1.2 m*M*) or not (nonHYK; plasma β-hydroxybutyrate < 1.2 m*M*) or both together^1^ when the interaction between regression coefficient and HYK was nonsignificant^2^.

	Regression Coefficients		Interaction		HYK and nonHYK together		
	nonHYK	HYK	SE	*P*-value^3^	Slope	SE	*P*-value
C16:0							
d 1	2.37	1.58	0.76	0.32	1.54	0.40	<0.01
d 14	5.65	1.98	5.32	0.50	2.06	1.39	0.15
C18:0							
d 1	2.37	0.28	1.51	0.18	0.64	0.62	0.31
d 14	1.18	-3.54	2.36	0.15	0.08	1.20	0.95
C18:1							
d 1	0.60	0.81	0.42	0.59	0.75	0.14	<0.01
d 14	1.42	-2.61	0.87	0.01	−	−	−
C18:2							
d 1	0.56	-0.10	0.23	0.01	−	−	−
d 14	0.44	0.37	0.36	0.32	0.24	0.20	0.23

^1^Overall regression coefficient is only presented when there was a lack of significance for the interaction between HYK and plasma proportion.

^2^Number of paired observations at each timepoint within group: HYK d 1 (n = 9); nonHYK d 1 (n = 11); HYK d 14 (n = 10); nonHYK d 14 (n = 11).

^3^The *P*-value associated with the interaction of HYK and plasma FA proportion in a regression model where the liver FA profile was the response variable.

**Fig 5:**
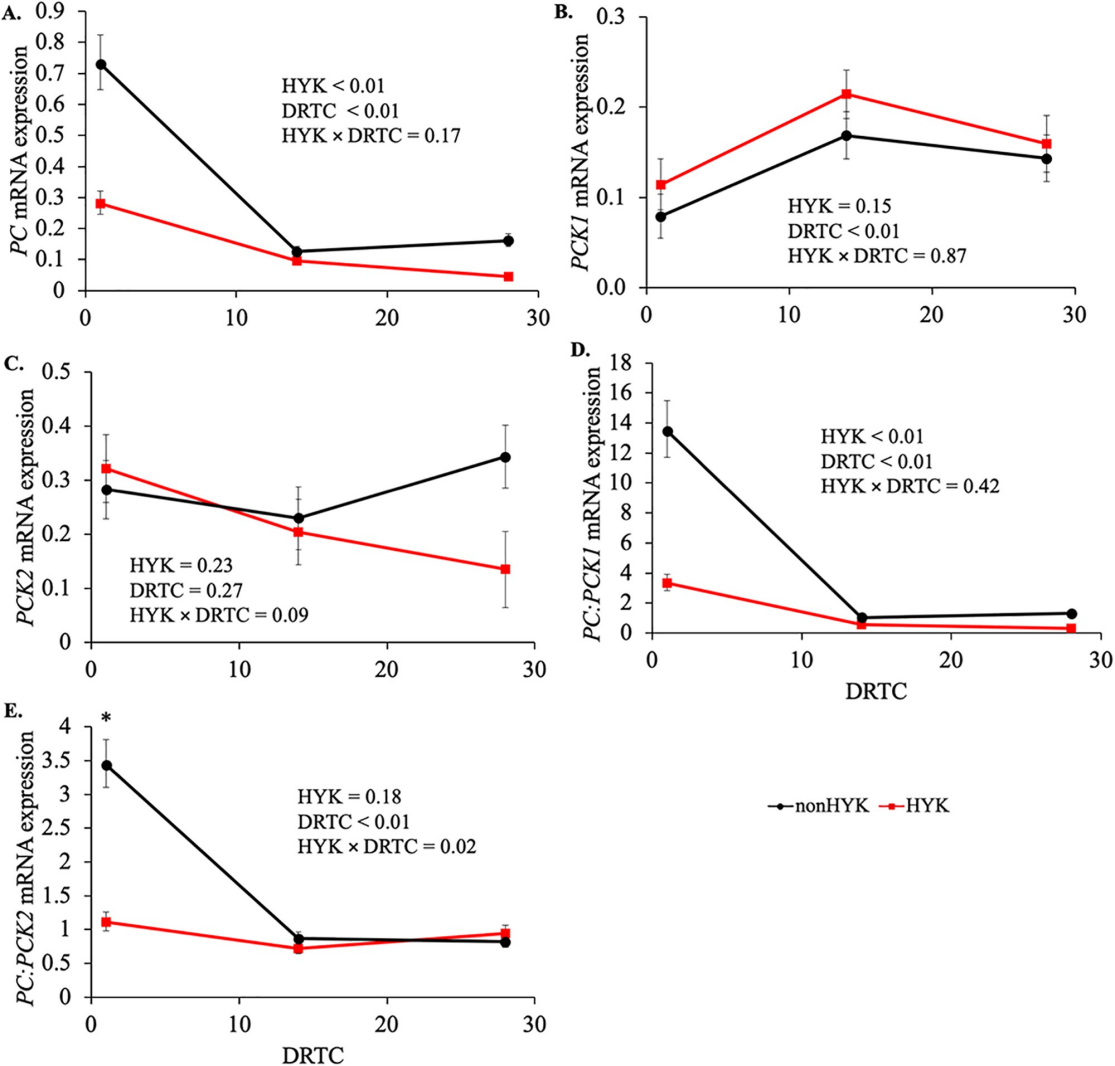
Hepatic expression of pyruvate carboxylase and phosphoenolpyruvate carboxykinase in cows diagnosed as hyperketonemic or not. Pyruvate carboxylase (*PC*; A), cytosolic phosphoenolpyruvate carboxykinase (*PCK1*; B), mitochondrial phosphoenolpyruvate carboxykinase (*PCK2*; C), *PC*:*PCK1* (D), and *PC*:*PCK2* (E) in cows diagnosed as hyperketonemic (HYK; plasma β-hydroxybutyrate ≥ 1.2 m*M*; red) or not (nonHYK; plasma β-hydroxybutyrate < 1.2 m*M*; black) during the postpartum period. Gene expression is expressed as arbitrary units (AU) relative to the reference gene. Expression of *PC*, *PC*: *PCK1*, and *PC*:*PCK2* were log transformed for analysis to achieve normal residuals and thus are reported using the 67% confidence interval. *PCK1* and *PCK2* were not log transformed for analysis and are reported using the SEM. Asterisks indicates difference between HYK and nonHYK within timepoint (*P* ≤ 0.05).

Cows later diagnosed with HYK had greater *PC* expression starting at 1 d postpartum (*P* < 0.01; Fig 5). There was not an effect of HYK detected on *PCK1* or *PCK2* expression (*P* > 0.15). In nonHYK cows, the *PC*:*PCK1* ratio was greater throughout the postpartum period (*P* < 0.01) and at d 1 for *PC*:*PCK2* (*P* < 0.01) compared with HYK cows. Gene expression of *PC* within HYK and nonHYK cow groups at d 1 was not related to the plasma NEFA concentration nor individual plasma FA proportions at 3 d prepartum or d 1 postpartum (*P* > 0.10; Table 6).

**Table 6:** Pearson correlations of plasma fatty acid (FA) proportions and non-esterified FA (NEFA) with hepatic gluconeogenic mRNA expression in cows diagnosed with hyperketonemia (HYK; plasma β-hydroxybutyrate ≥ 1.2 mmol/L) or not (nonHYK; plasma β-hydroxybutyrate < 1.2 m*M*)^1^.

	Correlations									
	NEFA		C16:0		C18:0		C18:1		C18:2	
Gene^2^	nonHYK	HYK	nonHYK	HYK	nonHYK	HYK	nonHYK	HYK	nonHYK	HYK
*PC*										
d 1	0.18	-0.02	0.53^†^	-0.10	-0.42	0.25	0.42	-0.20	-0.24	0.08
d 14	-0.49^†^	-0.25	0.20	-0.26	0.13	0.68*	0.16	-0.33	-0.38	0.19
d 1 with -3^3^	−	−	-0.16	-0.23	-0.22	-0.17	-0.02	0.10	0.42	0.00
*PCK1*										
d 1	0.29	0.44	0.30	0.43	-0.19	0.78*	0.17	0.44	-0.21	-0.72*
d 14	0.06	0.42	0.26	0.13	-0.52^†^	-0.02	0.31	0.38	0.28	-0.15
d 1 with -3	−	−	-0.35	0.04	-0.29	0.26	-0.20	-0.11	0.29	-0.34
*PCK2*										
d 1	0.14	-0.20	0.20	0.19	-0.30	0.53^†^	0.01	0.09	0.03	-0.38
d 14	-0.43	-0.05	0.13	-0.14	-0.01	0.23	0.14	0.03	-0.15	0.16
d 1 with -3	−	−	-0.25	-0.43	-0.16	0.67*	-0.03	-0.55^†^	0.07	-0.09

^1^Number of observations at a given timepoint and group: nonHYK d 1 (n = 14); HYK d 1 (n = 11); nonHYK d 14 (n = 12); HYK d 14 (n = 12); HYK d -3 (n = 10); nonHYK d -3 (n = 13).

^2^Pyruvate carboxylase: *PC*, cytosolic phosphoenolpyruvate carboxykinase: *PCK1*, mitochondrial phosphoenolpyruvate carboxykinase: *PCK2*

^3^Correlation of *PC* expression on d 1 with FA proportions at d 3 prepartum

*indicates *P* ≤ 0.05;

^†^ indicates 0.05 < *P* ≤ 0.10.

## Discussion

Many changes in metabolism and nutrient partitioning occur during the peripartum period to allow cows to meet the demands of lactation. The FA mobilized from adipose tissue play a necessary role as energy substrates, but they also regulate gene expression and cell signaling, which could contribute to shifts in nutrient partitioning postpartum in the presence or absence of metabolic disorders. Although inducing postpartum fatty liver through dietary manipulations has previously demonstrated shifts in plasma and liver FA profile in cows with fatty liver, the primary goal of this project was to understand how these changes in FA profile could impact hepatic metabolism of FA for storage or complete oxidation. In order to answer this question, plasma and liver FA profiles and hepatic gene expression had to be quantified in postpartum cows with or without HYK. Prepartum energy intake of animals in this experiment was greater than maintenance to increase HYK incidence of the study population (compared to the research herd average) to be comparable to that observed in the industry. This was a part of a larger study that included a postpartum dietary intervention that reduced BHB, plasma NEFA, and improved feed efficiency [15]. The subset of cows used for this study were randomly selected and include cows from each postpartum dietary treatment within the HYK and nonHYK groups. During data interrogation of the outcome variables examined in the current study, postpartum dietary intervention impacted DMI and although it interacted with time to affect pre- and postpartum intake, it did not interact with HYK status. Postpartum dietary treatment did not affect FA composition in plasma or liver. When included as a random effect with HYK, the covariance was 0 indicating it did not explain significant variation (see Caputo Oliveira et al., 2019 [15] for discussion of full dietary treatment effects).

The increased postpartum BHB and NEFA observed in HYK cows was reflective of the increased BCS loss in the immediate postpartum period (d 1 to 14). Previous studies have linked greater loss of BCS during pre- and postpartum periods [11, 27] and increased postpartum NEFA [28] with increased circulating BHB concentration postpartum. In this experiment, the decreased prepartum DMI of HYK cows caused a slightly less positive EB prepartum compared with their nonHYK herd mates, but HYK did not significantly affect EB postpartum. The decreased DMI in HYK cows agrees with previous research [29, 30], although due to the difference in BW between HYK and nonHYK cows in this study, we did not observe differences between DMI per BW of groups. It is evident that even when offered similar prepartum diets, cows will naturally differ in their propensity for postpartum HYK onset.

The major FA in adipose tissue are C16:0, C18:0, and C18:1 [8–10] and are expected to be increased in plasma postpartum as cows mobilize FA from adipose tissue, as was observed with increased postpartum plasma C16:0 and C18:1 relative to prepartum plasma in both HYK and nonHYK cows in the current study. Although both groups mobilized adipose tissue, the greater BCS loss and elevated NEFA of HYK cows would suggest that FA derived from adipose tissue would be further increased in plasma compared with the increase nonHYK cows experience. This is supported by the interaction between time and HYK diagnosis on plasma C18:1 which resulted in a significant increase in plasma C18:1 at d 3 postpartum in cows diagnosed with HYK; however, plasma C16:0 and C18:0 were not different between groups. Proportions of plasma C18:2 were decreased in all cows postpartum, and further decreased in cows diagnosed with HYK, as would be expected due to the dilution by other FA entering the bloodstream derived from adipose tissue. Although DMI was less in HYK cows, differences in plasma C18:2 is not likely due to reduced dietary contributions given that biohydrogenation is nearly complete in cows fed diets without added unsaturated FA [31]. Interestingly, plasma proportion of C18:0 in both groups of cows was decreased postpartum despite its presence in adipose tissue. Prior work has demonstrated that subcutaneous adipose tissue FA profile does not change across the transition period [8], making it unlikely that preferential mobilization plays a major role in differential FA metabolism, at least within the subcutaneous depots. Relative to C16:0 and C18:1, C18:0 is present in lower proportions in subcutaneous fat, but at a greater proportion in internal fat stores [9, 10] which are also mobilized during the peripartum period [32]. It is unknown if the difference in FA profiles and metabolic activity between adipose tissue depots [33] may impact the circulating FA profile or preferential mobilization and metabolism. The differential pattern of plasma C18:0 compared with C16:0 and C18:1 suggests that there may be differences in their metabolism postpartum.

The proportion of NEFA taken up by the ruminant liver seems to be constant across a range of concentrations [34–37]. There were no significant effects of HYK on liver FA profiles or liver TG. Historically, it was suggested that HYK and fatty liver often occur together [1, 2]; however, this may not be the case in more recent research presented here and elsewhere [38]. Alternatively, we could be lacking statistical power to detect the difference in liver TG between HYK and nonHYK cows (HYK × time, *P* = 0.12). As hypothesized, there were significant relationships between plasma and liver proportions of individual FA; however, these relationships varied across FA and HYK diagnosis. Liver and plasma proportions of C16:0 and C18:1 were related at 1 d postpartum. The relationships between liver and plasma C18:1 on 14 d postpartum, and C18:2 on 1 d postpartum, were impacted by HYK. It has been observed that C18:2 is preferentially retained in liver [39], perhaps due to its importance as an essential FA. We are uncertain what might be the cause of the effect of HYK on the relationship between plasma and liver C18:1 at d 14, but it is worth noting that for both interactions there are a few datapoints contributing heavily to the interaction. Research in sheep has suggested that uptake of specific FA by the liver varies with the quantity of FA available [40], which suggests there may be an influence of preferential uptake in bovine.

Interestingly, the proportion of C18:0 was not significantly related between plasma and liver in either the HYK or nonHYK group. Research in dairy cows with fatty liver has shown that total C18:0 content of liver TG did not increase postpartum, even when C18:1 and C16:0 did [8]. The differential hepatic storage and plasma accumulation of C18:0 compared with C16:0 and C18:1 suggests preferential uptake, release, or metabolism by at least one pathway within the body. Hepatic uptake of C18:0 has been demonstrated to be lower than that of C16:0 or C18:1 in sheep [40, 41], hamsters [42], and rats [43]. In rats, hepatic C18:0 derived from chylomicrons has been shown to be preferentially directed towards phospholipids, while C16:0 is incorporated into liver TG [43]. If either of these were the case, we might expect a relative increase in plasma C18:0 compared with those FA taken up to a greater degree (i.e., C16:0 and C18:1); however, the opposite was observed. It also may be possible that in bovine, the liver preferentially oxidizes C18:0. Considering other possible end fates within the body, 18 carbon FA are taken up by the mammary gland in similar proportions to 16 carbon FA in midlactation cows fed diets without supplemental fat [44] and transition cows [45]. Fatty acids are also an energy source to muscle tissue which could impact the circulating FA concentration and profile, but this utilization has not been explored in dairy cows to our knowledge.

Hepatic gene expression was of interest in this research to examine if differences in ketogenesis and lipid storage between HYK and nonHYK cows was reflective of genes that are used to suggest relative complete oxidative capacity. Given the anaplerotic role of *PC* and cataplerotic role of *PCK* to maintain the oxaloacetate pool, the proportion of these two genes represents a suggestion of the relative capacity for complete oxidation of acetyl-CoA in the liver [7]. The presence of compartment-specific isoforms, *PCK1* and *PCK2*, may allow for substrate-specific metabolism. Primarily, *PCK1* is responsive to the transition period and dietary treatments, and many TCA intermediates can be shuttled across the mitochondrial membrane [6]. It is interesting that *PCK2* tended to differ by HYK status; however, it is clearly the change in *PC* that drove the decreased *PC*:*PCK1* and *PC*:*PCK2* in HYK cows at 1 d postpartum. While gene expression is only suggestive, in the case of these two genes, mRNA expression parallels enzyme activity [13, 46, 47] and the data may suggest that the oxaloacetate pool, and thus the capacity of the liver for complete oxidation of acetyl-CoA at calving, may be greater for cows that do not later develop HYK. This is supported by other research demonstrating decreased activity and expression of various hepatic proteins involved in FA oxidation, such as acyl-CoA synthetase long chain, carnitine palmitoyl transferase II, and 3-hydroxy acyl-CoA dehydrogenase [48, 49] and decreased content of hepatic oxaloacetate [48, 50] at the time of diagnosis in cows with HYK compared with nonHYK cows. To our knowledge, this experiment is the first to demonstrate that a change in indicators of complete oxidative capacity occurs prior to HYK development. In this study, the earliest diagnosis of HYK (plasma BHB ≥ 1.2 m*M*) was 3 d postpartum with the average being 9 ± 5 d. Combined with an increase in NEFA, decreased complete oxidative capacity could contribute to increased ketogenesis and liver TG in HYK cows. This ultimately would lead to greater liver TG and circulating BHB concentrations in cows later diagnosed with HYK, as we observed. Further research is needed to quantify the oxaloacetate pool, PC activity, and complete oxidative capacity prior to HYK onset to fully understand the impact of shifts in *PC* on hepatic metabolism.

As previous work has indicated that *PC* is regulated by NEFA concentration and profile [12, 25] correlations between *PC* expression and FA concentration and profile were examined to explore if individual FA were related to the differential expression of *PC* at 1 d postpartum. Although mRNA turnover in liver is on the order of hours [51], we also evaluated whether prepartum FA (3 d prepartum) were correlated with 1 d postpartum *PC*. We did not observe any significant correlations between either NEFA nor individual FA with *PC* in either case. The lack of relationship between *PC* and FA suggests that the difference in *PC* between HYK and nonHYK cows is not due to the differences in circulating FA concentration or profile observed between HYK and nonHYK cows, although this does not exclude the likelihood that FA concentration and profile are contributing to regulation of *PC* across the transition to lactation period. Previous observations of *PC* response to FA in cell culture used a greater FA concentration than observed in the current study. In fact, White et al., 2012 only observed increase of *PC* mRNA expression when MDBK cells were exposed to 1 m*M* compared to 0.5 m*M* of a FA profile representative of a cow with induced fatty liver [25]. Similarly, rat hepatoma (H4IIE) cells transfected with bovine *PC* promoter-luciferase constructs exposed to serum from feed restricted cows or serum from control cows with FA added had increased *PC* promoter 1 activity; however, the FA concentration in serum in that experiment was 1.3 m*M* [12]. Conversely, in the current study, *PC* mRNA expression was less at calving in cows that subsequently developed HYK. Concentrations of NEFA were greater in HYK cows but were still only 0.6 m*M* on average. Research in primary bovine hepatocytes suggests that small changes observed in FA profile, such as those observed between HYK and nonHYK cows, do not differentially regulate *PC* mRNA expression [22]. Although these findings do not exclude the possibility of regulation of *PC* by presence of FA or by larger shifts in concentration as observed previously [12, 25], it does support that differential response of *PC* within biological situations that elicit smaller shifts in FA variation may be due to additional regulatory aspects. Collectively, these data suggest that there may be alternative signaling molecules or regulatory metabolites in HYK cows at the time of calving eliciting the decrease in the expression of *PC*, independent of the FA present and future research should seek to elucidate this causative signal and discover if manipulation is plausible.

## Conclusions

In conclusion, hyperketonemia development in dairy cows was associated with decreased plasma C18:2 and increased plasma C18:1 proportion, but neither FA concentration nor profile were associated with differential regulation of *PC* at calving in the current work. The lack of relationship between plasma and liver C18:0 suggest the likelihood of preferential FA usage by the liver, although further work is required to determine mechanisms of action. Cows diagnosed with HYK had a significant reduction in *PC*:*PCK* mRNA expression at calving which may suggest a decreased hepatic capacity for complete oxidation prior to HYK development. This highlights metabolic shifts that occur at calving prior to subsequent onset of postpartum metabolic disorders and the potential importance of prepartum preventative interventions.

## Supporting information

S1 Fig (TIF)

S1 Material (XLSX)
