# Supplementary figures and images for: Hepatic pyruvate carboxylase expression differed prior to hyperketonemia onset in transition dairy cows
Source: PLoS One. 2020 Nov 9;15(11):e0241929. doi: 10.1371/journal.pone.0241929 (PMC7652276; doi:10.1371/journal.pone.0241929)

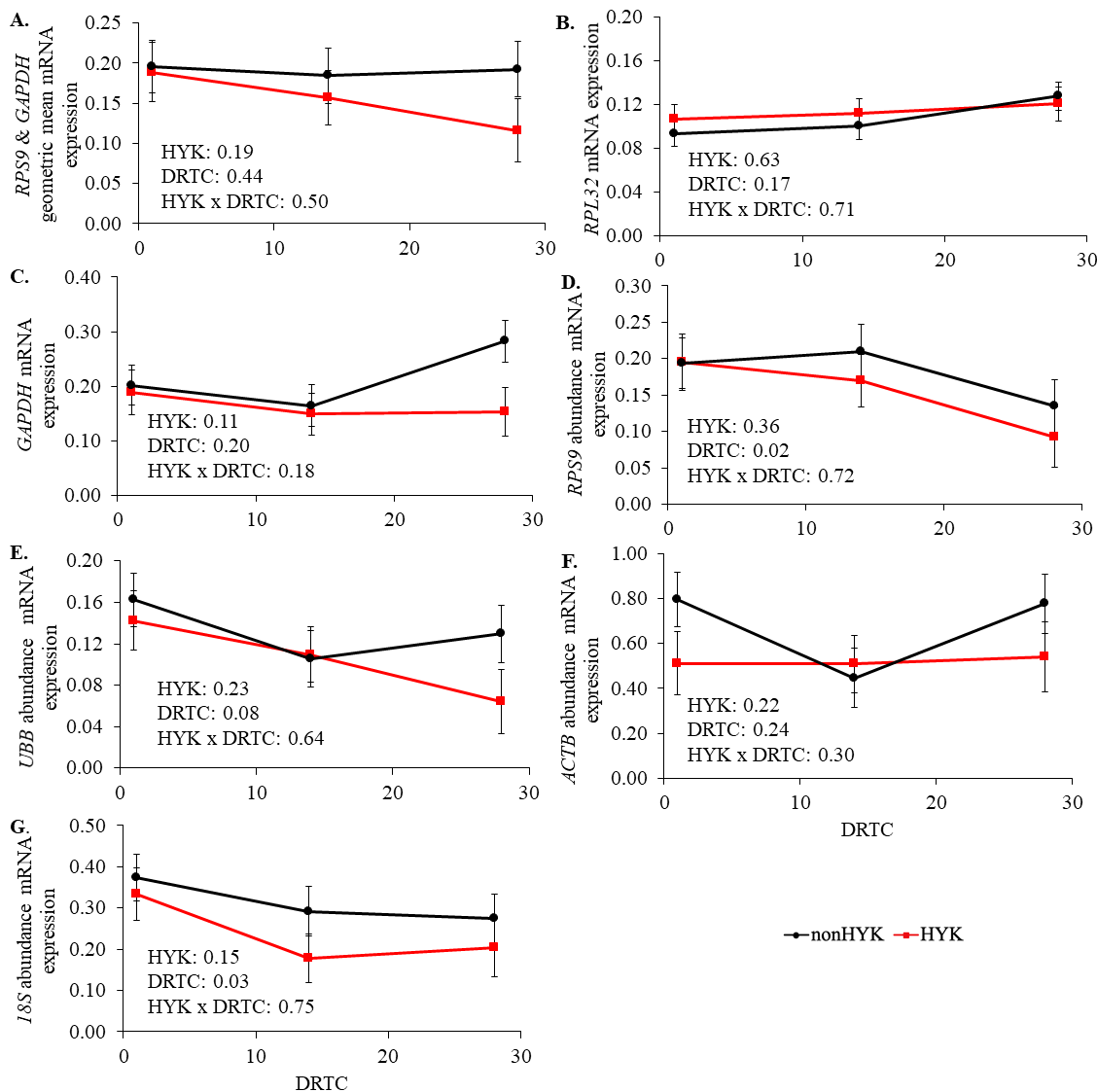

Supplement: S1 Fig — (TIF) [file pone.0241929.s001.tif]
